# Treatment Decisions in Advanced Disease: A Conceptual Framework

**DOI:** 10.4103/0973-1075.53509

**Published:** 2009

**Authors:** Bert Broeckaert, The Flemish Palliative Care Federation

**Affiliations:** Interdisciplinary Centre for the Study of Religion and Worldview, K.U. Leuven, Belgium; 1 J. Vander Vekenstraat 158, 1780 Wemmel, Belgium

**Keywords:** Palliative care, Euthanasia, Conceptual Framework

## Abstract

This English translation, made by a professional translator in close cooperation with the author and kindly proofread by Dr. Phil Larkin, follows the original text as closely as possible. However, though we thought it was wise to maintain the official (but not unproblematic) Dutch/Belgian definition of euthanasia in the original text (written for Belgian readers), the English texts offers a new and clearer definition of euthanasia. From the very beginning of the Belgian euthanasia debate in 1999, the Flemish Palliative Care Federation has chosen not to stay on the sideline, but to take an active part in the discussion and formulate recommendations based on our expertise and experience. Time and again we have pointed out that the ethical issues at the end of life are not just restricted to those of euthanasia. We have found that there is still much confusion about, for example, the difference or the boundary between pain control and euthanasia or between euthanasia and withholding life-sustaining treatment. Therefore, we thought it appropriate to put the following conceptual framework with regard to treatment decisions in advanced illness forward.

## INTRODUCTION

In this text, we would like to offer a typology about the different kinds of treatment decisions that can be taken in advanced stages of life-threatening illness. In other words, it is about the different ways medicine can help and support patients with advanced disease. We do not do this because we are keen on classifications, but because each kind of treatment decision brings about specific ethical issues which can be misunderstood when no clear boundaries and differences have been set.

We distinguish three major categories of treatment decisions in advanced disease [Table 1], where choices are with regard to:

**Table 1:** Ethical decisions option in advance stage of the disease

	Pain control	Palliative sedation	Euthanasia
Intention	Symptom control	Symptom control	Terminating life
Treatment	Administering as much medication as needed to control the pain (proportionality)	Administering as much medication as needed to control the symptom (proportionality)	Administering as much medication as needed to terminate life
Result	Life-shortening effect is exceptional (a lifelengthening effect is not)	Life-shortening effect is exceptional	Terminating life (by definition)

1. curative or life-sustaining treatment: is such a treatment initiated or withheld, continued or withdrawn?
2. palliative treatment and symptom control: all treatments aimed at maximizing, in an active way, the incurably ill patient's quality of life and comfort.
3. euthanasia and assisted suicide, where lethal medication is purposefully administered.

We will look into each of these categories further in this text. Our aim with this is not to present an elaborate ethical evaluation, but just to clarify the different concepts. From the fact that we mention and describe a certain act it cannot be deduced that that we approve of it or advocate it.

Our typology is not focused on life shortening, and does not take the life-shortening effect of the decisions as the common denominator. We deliberately opted not to do this. The classical euthanasia typology (indirect/direct, active/passive…) and the Dutch ‘medical decisions concerning the end of life’ typology deduced from it, do take this focus. This is wrong in our view, since such an approach leads to a one-sided and skewed perception of the delicate ethical choices to be made at the end of life.

Already the fact that we are dealing with ethical rather than purely medical decisions, which therefore should not be left to physicians alone, makes the term ‘medical decisions concerning the end of life’ inappropriate. Another reason for rejection of this term is precisely its one-sided focus on life shortening – one looks at reality from a euthanasia-perspective. It is, however, of utmost importance that we do not just look at life-shortening acts, but also at life-lengthening actions. Otherwise, we lose sight of, for instance, the whole issue of futile treatment (cf. infra).

Further, many decisions at the end of life have nothing or little to do with life shortening or life lengthening. The essential feature of pain control and palliative sedation is not the fact that they are life-shortening (or life-lengthening) and the discontinuing of an ineffective curative treatment does not have a life-shortening effect.

Sometimes our acts do have a life-shortening or life- lengthening effect, and this should indeed be involved in the deliberation. However, this is only one element in a much broader discussion about dignified living and dying: palliative care is in accordance with WHO's definition neither focussed on life lengthening, nor life shortening, but on the quality of life of the patient and his/her family. Therefore, it is out of question that because of a most likely unintended, but actual ‘euthanatic prejudice’, treatment decisions in advanced disease should only or chiefly be seen as life-shortening.

## (FORGOING) CURATIVE OR LIFE-SUSTAINING TREATMENT

When a life-threatening disorder, despite a treatment intended to cure or to sustain life, evolves in an adverse way, physician and patient face a number of difficult choices. Shall all or some treatments be continued in the hope that the tide will eventually (temporarily) turn? Shall other, more radical and risky treatments be chosen? Or shall all attempts to stop or curb the course of the illness be ceased? A first series of decisions to be made thus relates to curative and life-sustaining treatment. In many cases, the decision to withhold this treatment or not, will influence the patient's quality of life and time that he/she still has left.

### (Non)-treatment decisions

Choices made with regard to curative (aimed at recovery) or life-sustaining (aimed at sustaining life) treatments in an advanced stage of a life-threatening illness are rarely simple. Only rarely it is absolutely clear what the real chances for success are and what should be considered meaningful (100% meaningful) and futile (100% futile). What one considers a dignified and meaningful life, depends to a large extent on one's values and appreciations. There are also hardly any objective measures for weighing life lengthening or chance for possibly only partial recovery against quality of life. Therefore, it is imperative to involve the patient (or, if it is a patient who is unable to give informed consent, his/her relatives) as much as possible in these decisions.

Decisions on curative or life-sustaining treatments can turn out in two ways. Either one chooses to withdraw or withhold a certain treatment, since this treatment is no longer considered to be meaningful or effective in the given circumstances. Or one chooses to continue or initiate a treatment aimed at recovery or life sustainment – which does indeed pre-suppose the informed consent of the (competent) patient (cf. infra 1.2). This is a perfectly correct decision when the treatment stands a fair chance to be effective and is considered meaningful. However, a decision to continue or initiate a treatment can also amount to futile treatment. A treatment is considered futile when in the given circumstances it can no longer be considered meaningful or effective.

It is imperative to bear both options, initiating and withholding, withdrawing and continuing, in mind and not just to focus on one of them and then turn this option into a problem or put this option forward as the one and only choice. Withholding a life-sustaining treatment can sometimes be an ethical obligation; while it can be the opposite in other cases. Therefore, it would be wrong to just focus on non-treatment decisions when we are talking about decisions regarding curative or life-sustaining treatments.

It would be equally wrong to just look at non-treatment decisions as life-shortening treatments. Not to artificially prolong the dying process can not just be considered as life shortening. It is simply wrong to suggest that the standard should be to prolong a person's life (or rather: dying) to a maximum by means of the most sophisticated technology that exists, even if there is no quality of life left, and to speak of life shortening as soon as one deviates from this ‘standard’. When death is inevitably near, respecting the natural dying process where one refrains from drastic life-sustaining techniques will often be considered to be the most humane option.

Moreover, numerous non-treatment decisions have nothing to do with life shortening (or rather the non-lengthening of life). It is often about treatment which is no longer effective, which do not lead to the wished for recovery or lengthening of life. These treatments often have a serious and meaningless negative impact on the patient's comfort and quality of life, since they cause discomfort and suffering. Futile treatment continues to be a real problem, the extent and tragic consequences of which cannot be overrated, and which does deserve all possible attention.

Since many non-treatment decisions are foremost ethical decisions (about values and appreciations), the patient (or, if it is a patient who is unable to give informed consent, his/her relatives) has to be involved as much as possible in the decision-making process. This also holds for the so called DNR (‘do not resuscitate’)-codes, where it is decided beforehand to refrain from certain actions (e.g. resuscitation or administration of antibiotics). In this situation too, unilateral decisions taken without consultation are only rarely appropriate.

- Initiating or continuing a curative or life-sustaining treatment
- Non-treatment decision: “withdrawing or withholding a curative or life-sustaining treatment, because in the given situation this treatment is deemed to be no longer meaningful or effective”

### Refusal of treatment

In this context, it is important to point out that a medical treatment can also be withheld or withdrawn because of a fundamentally different reason than the aforementioned appraisal. There exists a growing international consensus which says that when a competent patient refuses a treatment, one should respect this refusal. Applied to health care, the right for physical integrity (a human right) implies that medical actions can only be performed when the patient gives his/her consent for these actions (cf. the Belgian Patients’ Rights Act).

When a patient refuses or withdraws his/her consent for a treatment (and this can also be done in an advance directive), one should respect this, even when this refusal, according to the health care providers, can be pernicious to the chance of recovery or the patient's quality of life, or can even inevitably lead to an early death. Healthcare providers do have the right and the duty to express their concern towards the patient and to point out the risks he or she takes. However, if the patient keeps on refusing, than this refusal should be respected.

“Withdrawing or withholding a curative or life-sustaining treatment, because the patient refuses this treatment”

## PAIN AND SYMPTOM CONTROL

When the treatment of a life-threatening illness is not successful and it is feared that the end is near, then the patient and his/her family are, of course, not just left to fend for themselves. Active care remains possible and necessary. The focus will,- and this is often a gradual process, shift from a curative and life-sustaining treatment to a palliative approach. And so, we are confronted with a second category of treatment decisions where we deal with the ethical questions and decisions with regard to pain and symptom control.

Palliative care no longer focuses on recovery or life sustaining measures. It is all about the patient's and his/her family's comfort. Through an inter-disciplinary and comprehensive approach, one tries to control symptoms in the best possible way and to offer the highest possible level of quality of life (physical, psychosocial and spiritual). With the required expertise, inter-disciplinarity and, if need be, the support of a specialised palliative care team, a great deal of suffering can be removed and a humane end of life becomes possible. In the following paragraphs, we are going to look into two kinds of symptom control which play a central role in the ethical discussion on the end of life: the medicinal treatment of physical pain and palliative sedation.

### Pain control

Even if palliative care fully acknowledges that pain comprehends much more than just physical pain and even if it tries to offer a solution for the patient's and his/her relatives’ ‘total pain’ by means of its inter-disciplinarity and a comprehensive approach, a lot of attention is paid to the treatment of physical pain. Physical pain often has a harmful effect on the quality of life, so that the given care, no matter how good, loses its sense and meaning if there is insufficient pain control.

Specialised palliative care has acquired a great deal of expertise in the field of pain control. In almost all cases, physical pain can be effectively controlled by means of adapted pain medication and adjuvant therapies. In this text, we will focus on the crucial medicinal treatment of pain and the major ethical questions evoked.

In the epidemiological and ethical research with regard to pain control, it would be wrong to only focus on those cases where heavy pain medication is administered. In this way, the most important ethical problem with regard to pain control is ignored -, the under-treatment of pain, which still causes that some incurably-ill patients die in simply inhumane circumstances. Inhumane circumstances which, tragically enough, could probably have been avoided with sufficient palliative experience and expertise.

Since there are still a number of misunderstandings about pain control which contribute to the aforementioned undertreatment we think it to be important to provide here too a clear definition. We define pain control in the following way:

“The intentional administration of analgesics and/or other drugs in dosages and combinations required to adequately relieve pain.”

The idea of proportionality (administering the medication and dosages needed) plays a central part in this definition. Typical in pain and symptom control is not only the physician's subjective intention (that is to say: treating a symptom, in this case, pain), but also the adequacy and proportionality of what is being done on an objective level (the act). One has to adjust the medication and dosages to the patient's actual or expected pain. What is the result of pain control? It is important to point out that, against all popular assumptions and suggestions, specialised experience and studies show that when pain control is done according to the rules, it, even when heavy medication is administered in high dosages, only very rarely has a life shortening effect. On the contrary, experience suggests that it quite often actually lengthens life.

These explanations illustrate that pain control on the one hand and euthanasia and assisted suicide on the other hand, cannot and should not be confused. They fundamentally differ on three levels: the intention (termination of life), the act (administering as much as is needed to terminate life) and the result (by definition life-shortening) are totally different when it comes to euthanasia or assisted suicide.

Therefore, we are explicitly opposed to phrases such as ‘raising painkillers’ or ‘intensifying pain control’. Their vagueness and ambiguity perpetuate and strengthen the confusion and abuse of the situation. For, they include both those cases where with good reason – due to the insufficiency of a former, lighter treatment- the medication or the dosages are carefully increased, and those other cases where something totally different is going on. Those who ‘intensify pain control’ with the main or side purpose of speeding up the end of life, are not at all concerned about the abovementioned and crucial adequacy or proportionality. They will often knowingly, specifically to shorten life, administer higher dosages and heavier medication than necessary to alleviate the pain, which results in a number of unwanted side effects as well like e.g. hallucinations, nausea and drowsiness.

Put more succinctly: here one is performing euthanasia and not pain control. The fact that this generally appears to be a ‘slower’ form of euthanasia does not change the actual character of this action. Such language (‘no problem: I will just intensify pain control’) puts pain control in a poor light, as pain control and euthanasia all become one and the same, and causes fellow-physicians, patients and their family to be afraid of the use of heavy pain medication, leading to under-treatment of serious pain as a tragic consequence.

### Palliative sedation

When, in very exceptional cases, it does not seem possible to sufficiently control certain physical or psychological symptoms with means which maintain patients consciousness, it can be decided within palliative care, together with the patient and his/her family –and with a competent patient of course not without his/her informed consent-, to perform palliative sedation. Palliative sedation implies that one purposefully and deliberately lowers the patient's level of consciousness to a level where one or several refractory symptoms can be sufficiently subdued. Refractory symptoms are symptoms which cannot be controlled by an experienced and specialised team. A refractory symptom is not the same as a difficult or complex (but with the necessary specialised expertise treatable) symptom. Therefore, it is generally indicated that a palliative sedation decision is preceded by specialised palliative advice.

Thanks to palliative sedation, a dignified dying process is even possible in very precarious circumstances. Palliative sedation can be mild or deep, continuous or intermittent (at intervals) and can be administered because of refractory symptoms of a physical and/or psychological or existential nature. If done properly palliative sedation has only in exceptional circumstances a life-shortening effect.

What about artificial nutrition and hydration?

The problem is obvious: those who are sedated can often no longer eat or drink normally. If this patient is not being artificially administered food and fluids, it gives rise to a situation which inevitably seems to lead to death. This impression does, however, need to be adjusted immediately. A vast majority of sedated patients are so close to death that the life-shortening effect of not artificially hydrating (let alone feeding) is practically zero. However, we did not include the withholding or withdrawing of artificial hydration in our definition and neither is it standard practice. When sedation is combined with the withholding of artificial nutrition and hydration, then it is not just sedation (inducing sleep) what is done, but it is *and* sedation *and* the withholding or withdrawing of artificial nutrition and/or hydration. This needs to be communicated in this way.

“The intentional administration of sedative drugs in dosages and combinations required to reduce the consciousness of a terminal patient as much as necessary to adequately relieve one or more refractory symptoms”

Palliative sedation is a special kind of symptom control, no more, no less. The physician's intention here is to fight the symptoms (and not to terminate life). Here too what is effectively done, at an objective level, needs to reflect this intention. In other words, in a field where dosages and combinations are crucial (if one administers too much, than there is a real danger of shortening the patient's life), the dosages and combinations administered have to be in proportion to the specific patient's suffering which one wants to alleviate. Adequacy and proportionality are again of central importance here.

Sedation which is administered according to the rules, is clearly not euthanasia: the intention (symptom control), act (you only administer what is necessary to control the symptom) and result (a life-shortening effect is exceptional) are totally different. A physician who claims to carry out palliative sedation, but in fact knowingly overdoses to shorten a patient's life is performing ‘slow euthanasia’, not carrying out palliative sedation. The sedation boundary is also crossed when the physician decides to speed up the dying process because the sedation takes longer than expected. Whoever administers more than what is necessary because he or she does not know what he or she is doing, is commiting medical malpractice. It is clear that in none of these cases we are dealing with palliative sedation.

## EUTHANASIA AND ASSISTED SUICIDE

With this third category we leave normal medical practice. This third and last kind of treatment decision is obviously an exceptional and controversial category. We would like to stress again that the fact that we mention or describe an act, does not mean that we approve of it or advocate it.

As opposed to pain and symptom control (where a life-shortening effect is only an exceptional side effect), euthanasia and assisted suicide by definition aim at shortening or terminating life: it is exactly the intention of the action of the person who acts to end or shorten the patient's life. Starting from this intention, one chooses the medication and determines the dosages (act). The result of euthanasia and assisted suicide is by definition the patient's death. We can distinguish three kinds of acts that belong in this category.

### Voluntary euthanasia

Withdrawing or withholding a life-sustaining treatment (e.g. artificial hydration or nutrition) is not euthanasia. In the case of euthanasia (voluntary/non-voluntary) it is always an active intervention, in this case the administration of lethal medication, and not a withdrawing or withholding which causes death. A second characteristic of euthanasia (again both voluntary and non-voluntary) is the fact that the lethal action is carried out by a person other than the patient himself.

Voluntary euthanasia implies that the patient himself requests a termination of life. This does of course imply that the patient is capable of requesting, in other words, that he is competent. Or that he has been competent, since sometimes euthanasia is not done on the basis of an actual request, but on the basis of a request written in an advance directive. The Netherlands and Belgium are the only two countries in the world which have an act on euthanasia that allows voluntary euthanasia (in certain circumstances and provided that certain procedures are taken into account, exceptionally also on the basis of an advance directive). However, a legally enforceable right to voluntary euthanasia –which would jeopardise the physician's professional and moral autonomy - is out of the question, also in Belgium and the Netherlands.

“The administration of lethal drugs in order to painlessly terminate the life of a patient suffering from an incurable condition deemed unbearable by the patient, at this patient's request”.

### Assisted suicide

The only and crucial difference between euthanasia and assisted suicide is the fact that with euthanasia, it is a person other than the person concerned who carries out the lethal action, while with assisted suicide, it is the patient who performs the action. The Belgian act on voluntary euthanasia does not mention a single word about assisted suicide; therefore, the legal status of assisted suicide in this country is obscure. In Switzerland and Oregon (USA) assisted suicide is allowed under certain circumstances, while euthanasia is not. Only the Netherlands allow both voluntary euthanasia and assisted suicide in the same way.

“Intentionally assisting a person, at this person's request, to terminate his or her life”.

### Non-voluntary euthanasia

Besides voluntary euthanasia and assisted suicide, we also have non-voluntary euthanasia: in these cases lethal medication is purposefully administered without the patient's request, and this is the difference with voluntary euthanasia. Disproportionally raising pain medication and/or sedatives (cf. supra) with the intention to speed up the end of life also belongs to this category and it does not matter whether this procedure takes a few minutes or a couple of days. Neither the Dutch, nor the Belgian act on euthanasia provides for this extremely delicate possibility.

*“The administration of lethal drugs in order to painlessly terminate the life of a patient suffering from an incurable condition deemed unbearable, not at this patient's request”*.

## CONCLUSIONS

Medicine and healthcare can help and support patients with advanced disease in several ways. In doing so, several choices can be made: some are widely accepted, others are a lot more controversial. Rather than expressing an ethical evaluation on this or other practices, this text intends to offer a conceptual framework, thus laying the necessary foundation of a meaningful ethical dialogue about a number of delicate issues. Without the conceptual and terminological explanations we have offered, we end up in a thick mist that blurs our vision and judgment and, due to lack of a common understanding of a number of basic terms, renders each ethical debate impossible.

Palliative care aims to offer the best possible quality of life to terminally-ill patients and their relatives. In order to do this, one often has to take delicate ethical decisions: with regard to curative or life-sustaining treatment, with regard to pain and symptom control, maybe also with regard to euthanasia or assisted suicide. It is of utmost importance that, in all these cases, one tries to be as careful as possible. The patient's voice should play a central role. Specialised advice and professional support is often required. To get this kind of advice and support, each care provider can turn to the palliative team of his/her own region or institution.(Please consult Federatie Palliatieve Zorg Vlaanderen [Flemish Palliative Care Federation], *Omgaan met euthanasie en andere vormen van medisch begeleid sterven* [Dealing with euthanasia and other end of life issues], 6^th^ September 2003.)

## TREATMENT DECISIONS IN ADVANCED DISEASE – A CONCEPTUAL FRAMEWORK

### (Forgoing) curative and/or life-sustaining treatment

- Initiating or continuing a curative or life-sustaining treatment
- Non-treatment decision: “withdrawing or withholding a curative or life-sustaining treatment, because in the given situation this treatment is deemed to be no longer meaningful or effective”
- Refusal of treatment: “withdrawing or withholding a curative or life-sustaining treatment, because the patient refuses this treatment”

#### Pain and symptom control

- Pain control: “the intentional administration of analgesics and/or other drugs in dosages and combinations required to adequately relieve pain.”
- Palliative sedation: “the intentional administration of sedative drugs in dosages and combinations required to reduce the consciousness of a terminal patient as much as necessary to adequately relieve one or more refractory symptoms”

#### Euthanasia and assisted suicide

- Voluntary euthanasia: “The intentional administration of lethal drugs in order to painlessly terminate the life of a patient suffering from an incurable condition deemed unbearable by the patient, at this patient's request”.
- Assisted suicide: “intentionally assisting a person, at this person's request, to terminate his or her life”.
- Non-voluntary euthanasia: “The intentional administration of lethal drugs in order to painlessly terminate the life of a patient suffering from an incurable condition deemed unbearable, not at this patient's request”.
